# The effectiveness and safety of Wu tou decoction on rheumatoid arthritis: A protocol for systematic review and/or meta-analysis

**DOI:** 10.1097/MD.0000000000029105

**Published:** 2022-03-18

**Authors:** Jeong-Hyun Moon, Won-Suk Sung, Seong-Kyeong Choi, Jung-Hyun Kim, Jin-woo Suh, Joo-Hee Kim, Byung-Kwan Seo, Seung-Deok Lee, Eun-Jung Kim

**Affiliations:** ^a^ *College of Korean Medicine, Dongguk University Graduate School, Seoul, South Korea,* ^b^ *Department of Acupuncture & Moxibustion, Dongguk University Bundang Oriental Hospital, Gyeonggi-do, Republic of Korea,* ^c^ *Department of Acupuncture & Moxibustion, Kyung Hee University Hospital at Gangdong, Seoul, Republic of Korea,* ^d^ *Department of Korean Neuropsychiatry, College of Korean Medicine, Sangji University, Gangwon-do, Republic of Korea,* ^e^ *Department of Acupuncture and Moxibustion Medicine, College of Korean Medicine, Sangji University, Gangwon-do, Republic of Korea,* ^f^ *Institute of Oriental Medicine, College of Korean Medicine, Dongguk University, Goyang-si, Republic of Korea.*

**Keywords:** meta-analysis, randomized controlled trials, rheumatoid arthritis, systematic review, Wu tou decoction

## Abstract

**Background:**

Rheumatoid arthritis (RA) is one of the common inflammatory diseases with arthritis due to a malfunction of the immune system. The treatments for RA include surgery, physiotherapy, occupational therapies, and medication. The representative treatment is medication and its usage has improved through several guidelines. However, it has some limitations and occurs adverse effects. Meanwhile, traditional Chinese medicine treatments have been used for RA treatment and Wu tou decoction (WTD) is one of them. Regardless of recent studies about WTD’s efficacy on RA, there has been no systematic review on this issue. Therefore, this review will focus on the effectiveness and safety of WTD on RA.

**Methods:**

The search for randomized controlled trial about WTD on RA will be performed using multiple electronic databases, manual searches, and the author’s e-mail if necessary. According to predefined criteria, randomized controlled trials will be selected and summarization will be performed by the data on study participants, result measurements, interventions, adverse events, and risk of bias. Disease activity score including effective rate, swollen joint count, tender joint count, morning stiffness will be primary outcome measures while blood test about RA including erythrocyte sedimentation rate, C-reactive protein, rheumatoid factors, and adverse events will be secondary outcome measures. We will perform meta-analysis by using Review Manager software, assess the risk of bias by Cochrane Collaboration “risk of bias” tool, and determine the quality of evidence by Grades of Recommendation, Assessment, Development, and Evaluation.

**Results:**

This study we will investigate the clinical evidence of the effectiveness and safety of WTD on RA.

**Conclusion:**

For the RA patients and clinicians, our study will be informative. It can be also a great help for the researchers and policy makers who concentrates on conservative management for RA.

**Trial registration number:**

INPLASY; INPLASY202220099

## 1. Introduction

Rheumatoid arthritis (RA) is one of the most common forms of inflammatory disease. It is an autoimmune disease that represents arthritis and occurs in the joints due to the malfunction of the immune system.^[1]^ It can occur at any age, but most often in the ages of 30 and 50.^[2]^ Several articles reported that the incidence rate worldwide reaches up to 1% and it has a prevalence of 0.3% to 1.1% in the European population and 0.1% to 0.5% in the Asian population.^[3-4]^

The diagnosis of RA is based on the classification criteria of American college of rheumatology /European league against rheumatism . It includes the followings; at least 1 joint must have clear swelling without specific cause, the production of autoantibodies including rheumatoid factors and antiarthritis protein antibodies, an abnormal rates of erythrocyte sedimentation rate and C-reactive protein, the duration of symptoms whether it is longer than 6 weeks or not.^[5]^

Currently, there has been no clear treatment for RA. There are several treatments including surgery, physiotherapy, and occupational therapies, but medications including nonsteroidal antiinflammatory drugs, glucocorticoids, and disease-modifying antirheumatic drugs (DMARDs) are commonly used for RA and the several clinical guidelines have updated and provided the recommendable usage in the various clinical practices.^[6,7]^ However, these medications are known to have some limited efficacy and occur adverse effects frequently. Adverse effects of DMARDs include thrombocytopenia, rash, and poor tolerability, as well as epileptic lung disease, liver damage, and the possibility of frequent infections, elevated cholesterol levels, and decreased blood cells.^[8,9]^

Traditional Chinese medicine treatments including decoction, acupuncture, and moxibustion have been used for RA treatment,^[10]^ and Wu tou decoction (WTD) is one of them. WTD consists of 5 herbs including *Radix Aconiti* (Wu tou), *Herba Ephedrae* (Ma huang), *Radix Astragali* (Huang qi), *Raidix Paeoniae Alba* (Bai shao), and *Radix Glycytthizae* (Gan cao). Regarding ingredients, WTD is referred to contain alkaloids, triterpene saponins, monoterpene glycosides, and flavones,^[11]^ which are known to play an effective role in suppressing the inflammatory response. Moreover, it moderates C-C chemokine receptor type 5 signaling pathways in macrophages and suppresses synovial hyperplasia and angiogenesis.^[12,13]^

In the arthritis model experiment, previous studies reported that WTD had inhibiting effects on the physiological activity of arthritis and the ability to alleviate the severity of collageninduced arthritis diseases.^[14]^ Some studies also reported that WTD could decrease the levels of interleukin -1β, interleukin -17, tumor necrosis factor-a, vascular endothelial cell growth factor, prostaglandin E2, and the percentage of CD4+ cells during increasing CD8+ cells in the arthritis rat model.^[15,16]^

Regardless of these impactful studies, there is limited clinical evidence of the effect and safety of WTD including systematic review (SR). Therefore, this review will focus on the effectiveness and safety of WTD on RA by analyzing the patient centered results, blood test, and following adverse events.

## 2. Methods

### 2.1. Study design

This SR and meta-analysis will follow the Preferred Reporting Items for Systemic reviews and Meta-Analyses Protocols 2015 statement.^[17]^

### 2.2. Ethics

Since there is no recruitment of patients and collection of personal information, ethical statement is not required.

### 2.3. Study registration

The protocol was registered in INPLASY (Registration number: INPLASY202220099).

### 2.4. Eligibility criteria

#### 2.4.1. Participants.

Patients diagnosed with RA will be included in the study. This study will cover all types of RA patients regardless of age and gender. However, patients who have osteoarthritis will be excluded.

#### 2.4.2. Types of interventions.

A randomized controlled trial (RCT) examining the effects of WTD on RA with a control group that includes other conservative treatments will be included. Conservative treatments include physiotherapy, occupational therapy, and medications such as nonsteroidal anti-inflammatory drugs, DMARDS, and glucocorticoids. The use of concomitant combination therapy with WTD should coincide with the experimental group and the control group.

#### 2.4.3. Type of studies.

This SR basically will include RCTs. This study will exclude the RCT which is unable to provide the description or used an unclear randomization method. Forms of non-RCTs including case reports, pilot studies, and SR will be excluded.

#### 2.4.4. Outcome measures.

Disease activity score including effective rate, swollen joint count, tender joint count, and morning stiffness will be primary outcome measures. Blood test about RA including erythrocyte sedimentation rate, C-reactive protein, rheumatoid factors, and adverse events will be secondary outcome measures.

#### 2.4.5. Language.

There will be no language restrictions in this review.

### 2.5. Information sources and search strategy

Searches will be performed on the following databases from the initiation to June, 2022: MEDLINE, Cochrane Library, China National Knowledge Infrastructure, CiNii, J-STAGE, Kore-aMed, Korean Medical Database, Korean Studies Information Service System, National Digital Science Library, Korea Institute of Science and Technology Information, and Oriental Medicine Advanced Searching Integrated System . The searches will be based on the language provided by each database. The search will continue in related literature materials, reports, and papers. If necessary, manual searches such as textbooks about RA and contacting the author’s email will be conducted (Table 1).

**Table 1:** Search strategy for the PubMed.

**No.**	**Search terms**
#1	Rheumatoid arthritis
#2	Rheumatoid OR arthritis
#3	#1 and #2
#4	Randomized controlled trial OR random* OR placebo
#5	Controlled clinical trial OR trial
#6	Wu Tou decoction
#7	Wu Tou tang
#8	#6 OR #7
#9	#3 and (#4 OR #5) and #8

### 2.6. Study selection

After 2 researchers have been trained on the designed qualification, each individual will independently screen the output based on the titles, abstracts, and full-text to exclude duplicates and irrelevant reports. And then, the 2 researchers will review the studies by reading their full-texts. Disagreement between the 2 researchers will be resolved through discussion. If not possible, a third reviewer will mediate it (Fig. 1).

**Figure:**
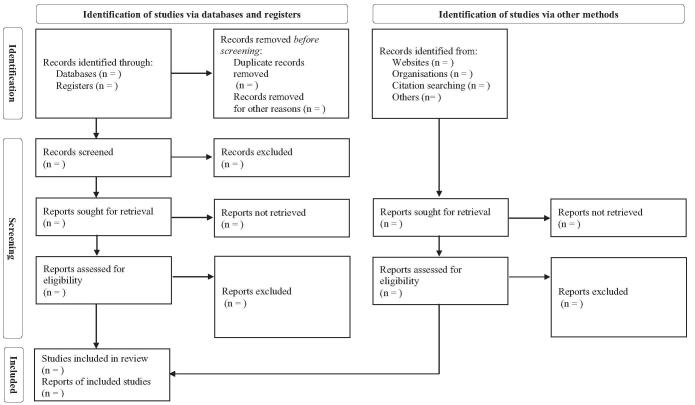
**Figure 1.** PRISMA flow diagram. PRISMA = preferred reporting items for systemic reviews and meta-analyses.

### 2.7. Data management

The results of the search will be edited using the widely used program Endnote X20.

### 2.8. Data extraction

The reviewers will extract information including patient characteristics, intervention between groups (process and duration), outcome measurement, the results, first author, publication year, and research quality. Disagreements will be mediated through discussion. For incomplete data, we will contact the author to try to obtain complete data. If it is insufficient, we will include the available data and describe it.

### 2.9. Data synthesis and analysis

The changes from baseline to intervention completion will be combined and the mean difference and 95% confidence intervals in same outcome measures, and the standardized mean difference and 95% confidence interval in different outcome measures will be calculated to estimate the effect with a random-effects or a fixed-effect model. Review Manager (Version 5.3; Copenhagen; The Nordic Cochrane Center, The Cochrane Collaboration, 2014) software for Windows will be use to perform a meta-analysis. Chi-squared and I-squared tests will be used to measure heterogeneity. The heterogeneity interpretation will follow these formats. 0% to 40%, 30% to 60%, 50% to 90%, 75% to 100% will described as unimportant heterogeneity, moderate heterogeneity, substantial heterogeneity, and considerable heterogeneity each. Potential variables would be found by sensitivity analysis or metaregression if necessary. Subgroup analysis will be conducted according to the main intervention of control group if possible. If it is impossible to create quantitative synthesis, narrative synthesis can perform as substitute. When there are over 10 trials included, funnel plot can be applied as assessment of publication bias. Quality of evidence will be rated by the Grades of Recommendation, Assessment, Development, and Evaluation.^[18]^

### 2.10. Risk of bias assessment

Two reviewers will independently assess the risk of bias using the “Risk of Bias” tool from the Cochrane Collaboration.^[19]^ This tool consist 7 areas: sequence generation, allocation concealment, blinding of participants and investigators, blinding of outcome assessment, incomplete outcome data, selective reporting, and other biases. The risk of bias for each domain is assessed as “low risk,” “high risk,” or “unclear risk.”

## 3. Discussion

RA is a disease that greatly affects an individual’s life in terms of pain and quality of life. Several conventional treatments and medications are used, but it has limitations with their insufficient efficacy and adverse effects. WTD has been suggested as an alternative option for RA, but no SR had been published. Our study will evaluate the clinical effectiveness and safety of WTD for RA treatment and provide useful resource for patients, clinicians, and researchers.

## Author contributions

**Conceptualization:** Won-Suk Sung, Eun-Jung Kim.

**Funding acquisition:** Joo-Hee kim.

**Investigation:** Moon Jeong-Hyun, Seong-Kyeong Choi, Jeong-Hyun Moon.

**Methodology:** Jung-Hyun Kim, Jin-woo Suh, Byung-Kwan Seo, Seung-Deok Lee, Eun Jung Kim.

**Project administration:** Joo-Hee Kim, Byung-Kwan Seo.

**Supervision:** Eun Jung Kim.

**Writing** - **original draft:** Moon Jeong-Hyun, Won-Suk Sung, Jeong-Hyun Moon.

**Writing - review & editing:** Won-Suk Sung, Seong-Kyeong Choi, Jung-Hyun Kim, Jin-woo Suh.
